# Supplementary material for: Olfactory memory representations are stored in the anterior olfactory nucleus
Source: Nat Commun. 2020 Mar 6;11:1246. doi: 10.1038/s41467-020-15032-2 (PMC7060254; doi:10.1038/s41467-020-15032-2)
Supplement: Supplementary file 1 — Supplementary Information [file 41467_2020_15032_MOESM1_ESM.pdf]

Supplementary Information

Olfactory Memory Representations Are Stored in the Anterior Olfactory Nucleus

Aqrabawi & Kim

Contents

1. Supplementary Figure 1-6.

2. Supplementary Table 1

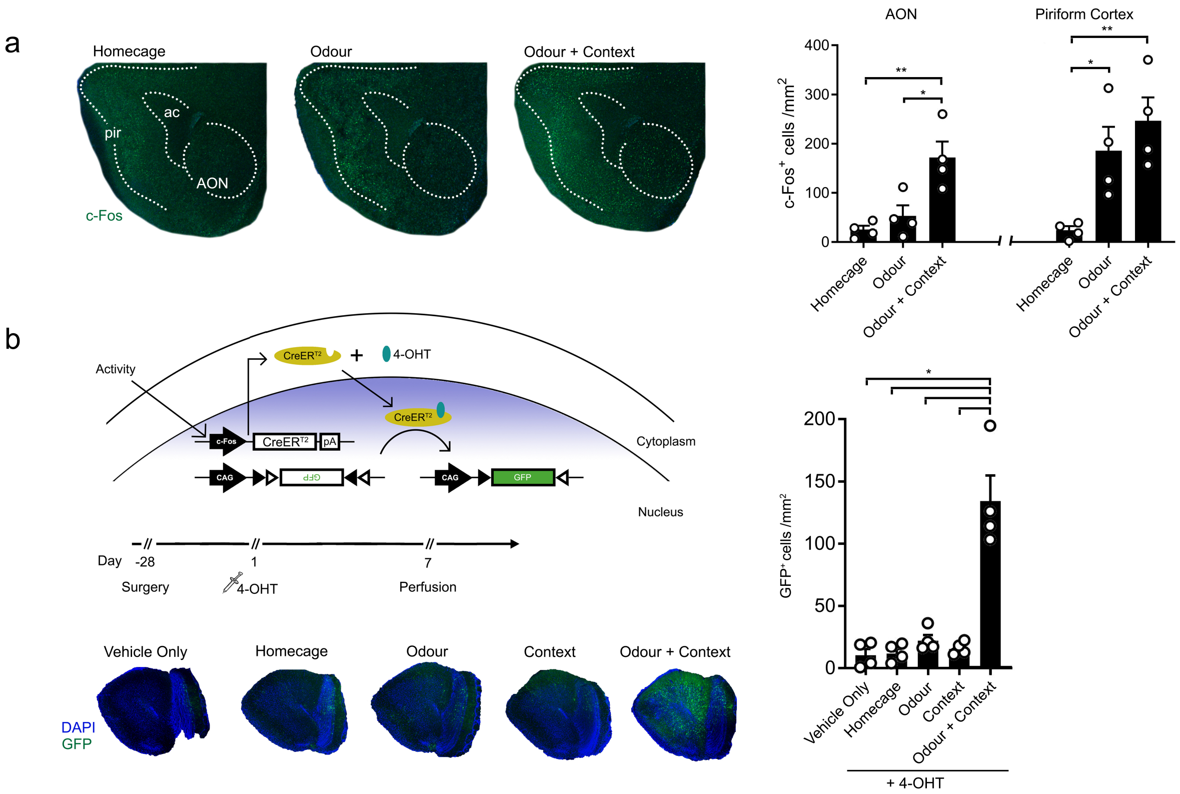

Supplementary Fig. 1 | Coincidence detection in the AON. a, Left: Posterior (bregma +2.0 mm) coronal sections depicting c-Fos expression within the AON and piriform cortex (pir). ac=anterior commissure. Right: Density of c-Fos$^+$ neurons measured in the AON and piriform cortex under different olfactory and contextual conditions [AON; each group, $n = 4$ mice, one-way ANOVA, $F(2, 9)=11.74$, $^{**}P=0.0031$; Piriform; each group, $n = 4$, one-way ANOVA, $F(2, 9)=8.490$, $^{**}P=0.0085$]. **b,** Left-top: Method for activity-dependent labelling of AON neurons with a GFP-reporter. Left-bottom: Representative coronal sections of the AON for each condition. Right: Quantification of the density of GFP$^+$ cells within the medial AON [each group, $n = 4$ mice, one-way ANOVA, $F(4, 15)=29.08$, $^{****}P<0.0001$]. Data are means ± SEM. Source data are provided as a Source Data file.

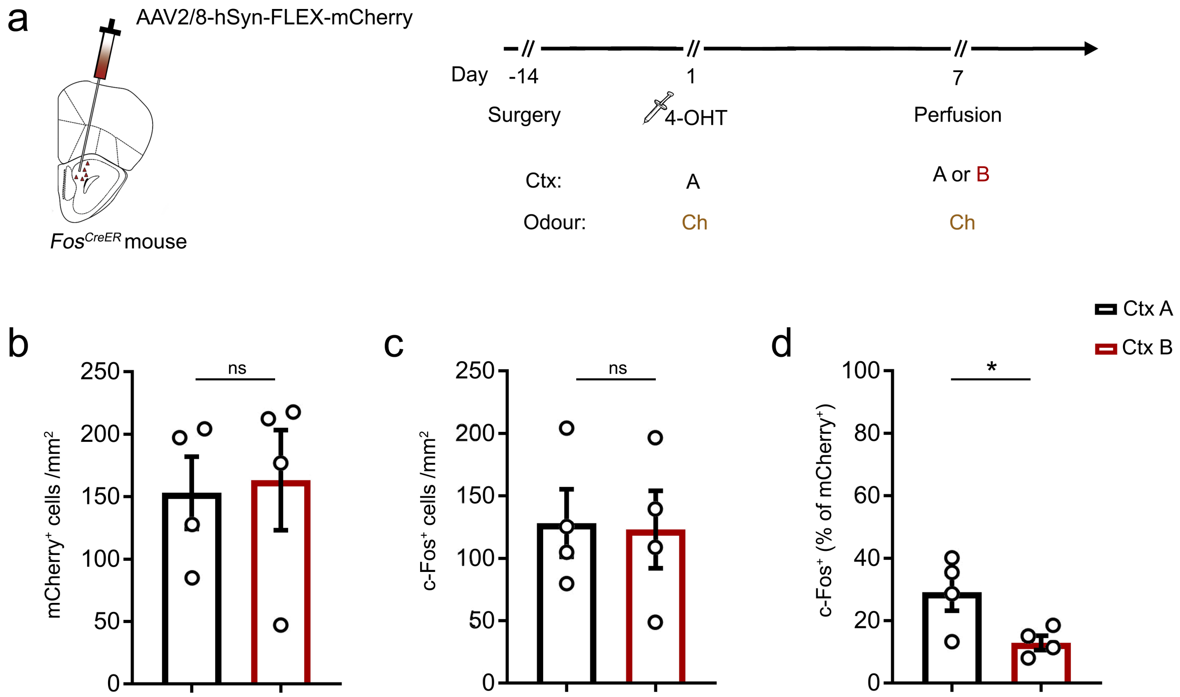

Supplementary Fig. 2 | Context-dependent changes in AON activity patterns. a, Experimental protocol. **b,** Density of labelled mCherry-positive cells in the medial AON [each group, *n* = 4 mice, two-tailed independent-samples *t*-test, *t*(6)=0.2036, *ns, P*=0.8454]. **c,** Overall level of activity measured using c-Fos expression [each group, *n* = 4 mice, two-tailed independent-samples *t*-test, *t*(6)=0.1255, *ns, P*=0.9043]. **d,** Corresponding reactivation rates in AON neurons [each group, *n* = 4 mice, two-tailed independent-samples *t*-test, *t*(6)=2.562, **P*=0.0428]. Data are means ± SEM. Source data are provided as a Source Data file.

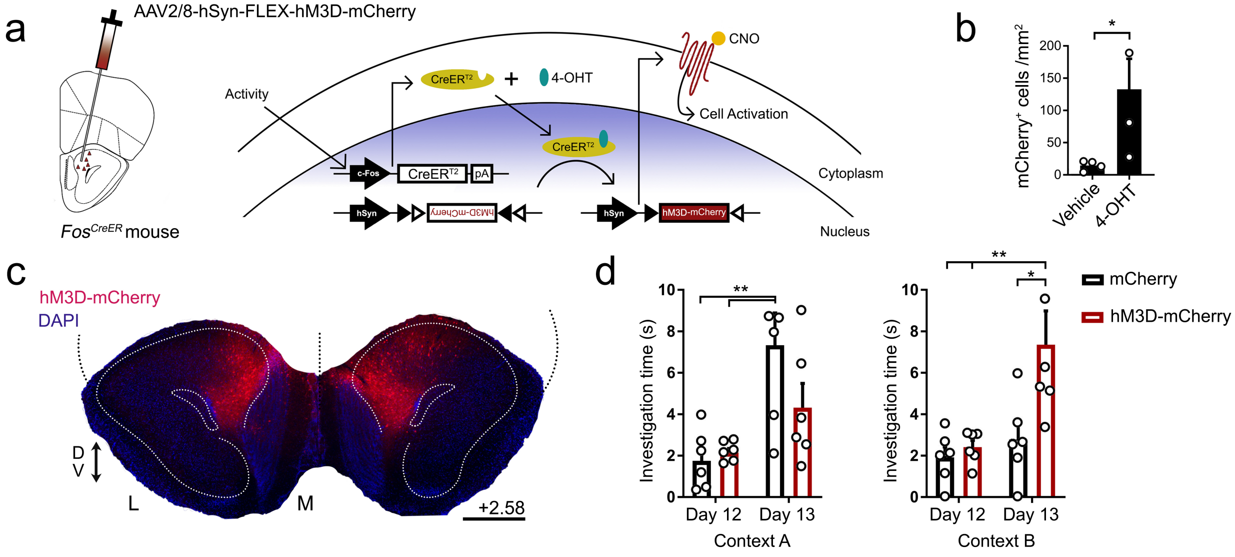

Supplementary Fig. 3 | Activating AON neurons involved in the context-driven odour recollection paradigm. a, Method for activity-dependent labelling of AON neurons with the excitatory hM3D-mCherry receptor for subsequent cell activation. **b,** The density of mCherry-positive cells within the medial AON observed following vehicle or 4-OHT injections [each group, $n = 4$ mice, two-tailed independent-samples t-test, $t(6)=2.486$, $*P=0.0474$]. **c,** Representative coronal section of the bilateral hM3D-mCherry expression in the medial AON. **d,** Behavioural expression of contextually-cued odour memory as measured by total investigation time of the odour source [Context A: each group, $n = 6$ mice, two-way ANOVA, main effect of group $F(1, 20)= 1.643$, $P=0.2146$; main effect of day $F(1, 20)= 14.39$, $**P=0.0011$; interaction between group and day $F(1, 20)=2.791$, $P=0.1104$; Context B: each group, $n = 6$ mice, two-way ANOVA, main effect of group $F(1, 20)= 7.019$, $*P=0.0154$; main effect of day $F(1, 20)= 9.208$, $**P=0.0065$; interaction between group and day $F(1, 20)=4.542$, $*P=0.0457$]. Data are means ± SEM. Source data are provided as a Source Data file.

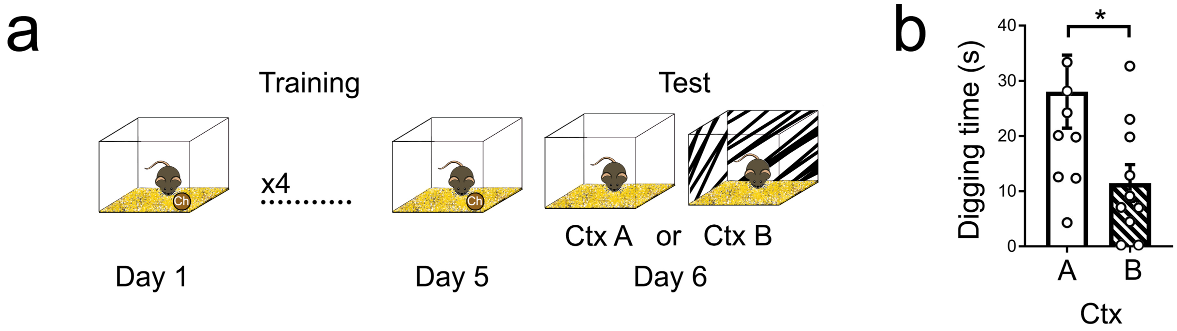

Supplementary Fig. 4 | Context-driven chocolate foraging. a, Protocol used for the formation and evaluation of a chocolate-context association. **b,** Total amount of time spent digging in a novel context or one previously paired with chocolate [each group, $n = 10$ mice, two-tailed independent-samples t-test, $t(18)=2.237$, $^*P=0.0382$]. Data are means ± SEM. Source data are provided as a Source Data file.

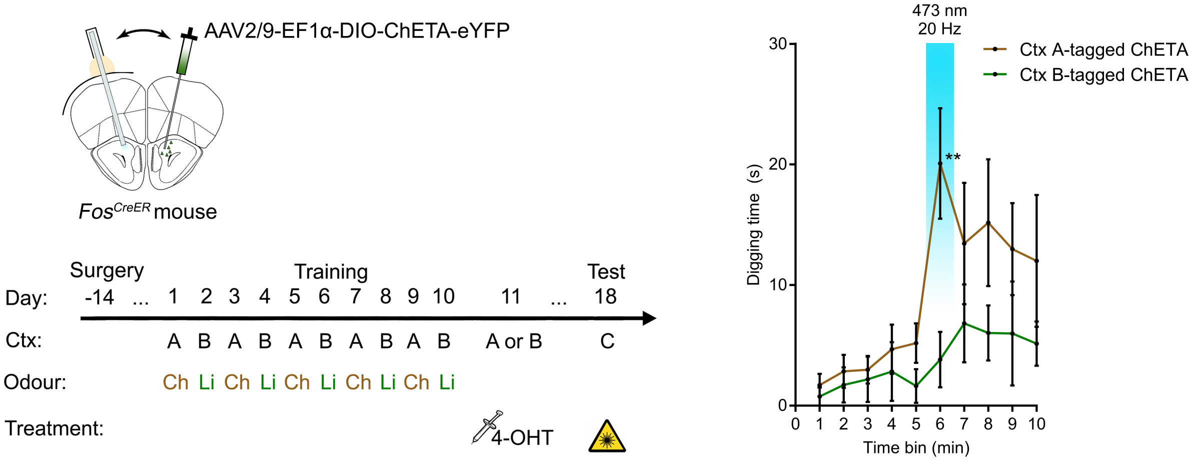

Supplementary Fig. 5 | Activating contextually-cued odour engrams. Left: Experimental protocol. Right: Animals injected with 4-OHT during exposure to the chocolate-associated context (Ctx A) showed a robust digging response upon ChETA-mediated activation of the AON. Activation of neurons tagged during exposure to the limonene-associated context (Ctx B) did not produce an increase in foraging behaviour [each group, $n = 6$ mice; two-way ANOVA, main effect of group $F(1, 100)= 15.74$, ***P=0.0001; main effect of time $F(9, 100)= 3.593$, ***P=0.0007; interaction $F(9, 100)= 1.272$, *ns*, P=0.2615]. Data are means ± SEM. Source data are provided as a Source Data file.

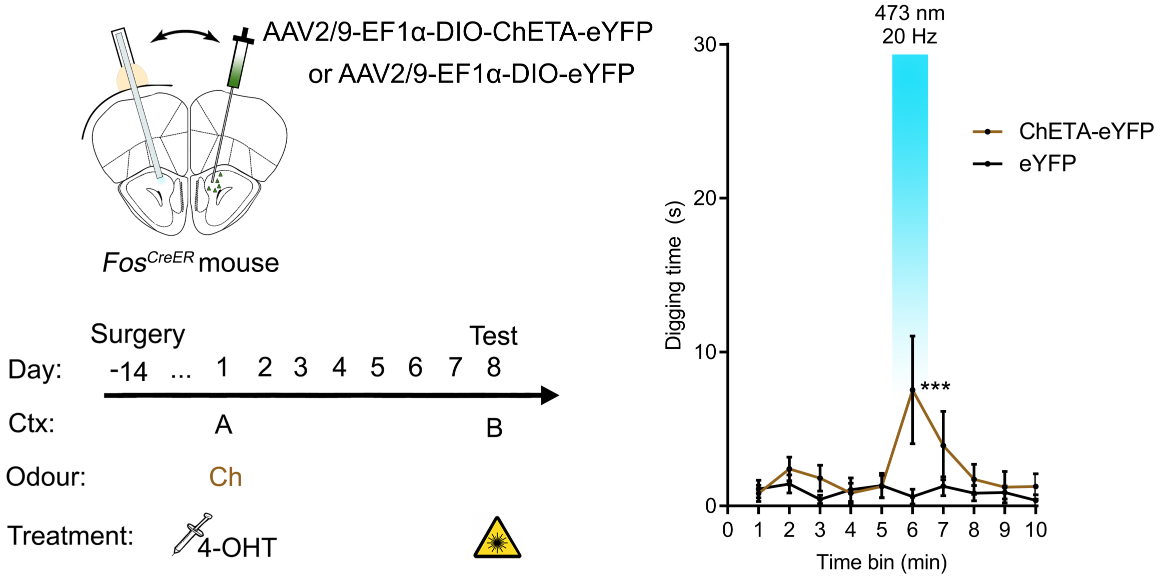

Supplementary Fig. 6 | Activating AON engrams formed following a single exposure to chocolate. Left: Experimental protocol. Right: ChETA-eYFP-expressing animals demonstrated significantly greater digging in response to blue light illumination, demonstrating a capacity for the AON to store odour information after one trial [each group, n = 6 mice; two-way ANOVA, main effect of group F(1, 100)= 7.253, ***P=0.0083; main effect of time F(9, 100)= 1.627, *ns, P*=0.1176; interaction F(9, 100)=1.797, *ns*, P=0.0780]. Data are means ± SEM. Source data are provided as a Source Data file.

Supplementary Table 1 | One-sample t-test (two-tailed) comparing preference scores to zero for the real-time place preference test depicted in Figure 2.

	Pre-Test Habituation			Test		
	YFP	Li-tagged ChETA	Ch-tagged ChETA	YFP	Li-tagged ChETA	Ch-tagged ChETA
**Sample size (*n*)**	10	10	10	10	10	10
**One sample t test**						
**Theoretical mean**	0	0	0	0	0	0
**Actual mean**	-0.063	-0.075	0.007	-0.048	-0.091	0.277
**Discrepancy**	-0.063	-0.075	0.007	-0.048	-0.091	0.277
**95% CI of discrepancy**	-0.2263 to 0.1003	-0.2238 to 0.07385	-0.2236 to 0.2376	-0.2003 to 0.1043	-0.3111 to 0.1291	0.00387 to 0.5501
**t, df**	t=0.8727 df=9	t=1.14 df=9	t=0.06867 df=9	t=0.7131 df=9	t=0.9353 df=9	t=2.294 df=9
**P value (two tailed)**	0.4055	0.2838	0.9468	0.4938	0.374	0.0474
**Significant (alpha=0.05)?**	No	No	No	No	No	Yes
