# Supplementary material for: Olfactory memory representations are stored in the anterior olfactory nucleus
Source: Nat Commun. 2020 Mar 6;11:1246. doi: 10.1038/s41467-020-15032-2 (PMC7060254; doi:10.1038/s41467-020-15032-2)
Supplement: Supplementary file 5 — Description of Additional Supplementary Files [file 41467_2020_15032_MOESM5_ESM.pdf]

Description of Additional Supplementary Files

File Name: Supplementary Movie 1
Description: **Chocolate engram activation drives foraging behaviour.** Representative footage of a mouse expressing ChETA-eYFP in AON neurons tagged during encoding of the chocolate odour. Animals were illuminated with yellow (589 nm) or blue (473 nm) light at specific 1 min intervals during a 20 min test. High (20 Hz) but not low (4 Hz) frequency stimulation is sufficient to elicit digging.

File Name: Supplementary Movie 2
Description: **Long-term activation of a chocolate engram.** Representative footage of a mouse expressing ChETA-eYFP in AON neurons tagged during encoding of a chocolate odour 60 days prior to testing. Animals illuminated with blue light (473 nm; 20 Hz) displayed robust digging behaviour.
