# Supplementary material for: Olfactory memory representations are stored in the anterior olfactory nucleus
Source: Nat Commun. 2020 Mar 6;11:1246. doi: 10.1038/s41467-020-15032-2 (PMC7060254; doi:10.1038/s41467-020-15032-2)
Supplement: Supplementary file 6 — Reporting Summary [file 41467_2020_15032_MOESM6_ESM.pdf]

nature research

Corresponding author(s): Dr. Junchul Kim; Dr. Afif Aqrabawi

Last updated by author(s): January 29, 2020

Reporting Summary

Nature Research wishes to improve the reproducibility of the work that we publish. This form provides structure for consistency and transparency in reporting. For further information on Nature Research policies, see Authors & Referees and the Editorial Policy Checklist .

Statistics

For all statistical analyses, confirm that the following items are present in the figure legend, table legend, main text, or Methods section.

n/a	Confirmed	
☐	☒	The exact sample size (*n*) for each experimental group/condition, given as a discrete number and unit of measurement
☐	☒	A statement on whether measurements were taken from distinct samples or whether the same sample was measured repeatedly
☐	☒	The statistical test(s) used AND whether they are one- or two-sided<br>*Only common tests should be described solely by name; describe more complex techniques in the Methods section.*
☐	☒	A description of all covariates tested
☐	☒	A description of any assumptions or corrections, such as tests of normality and adjustment for multiple comparisons
☐	☒	A full description of the statistical parameters including central tendency (e.g. means) or other basic estimates (e.g. regression coefficient) AND variation (e.g. standard deviation) or associated estimates of uncertainty (e.g. confidence intervals)
☐	☒	For null hypothesis testing, the test statistic (e.g. *F*, *t*, *r*) with confidence intervals, effect sizes, degrees of freedom and *P* value noted<br>*Give P values as exact values whenever suitable.*
☒	☐	For Bayesian analysis, information on the choice of priors and Markov chain Monte Carlo settings
☒	☐	For hierarchical and complex designs, identification of the appropriate level for tests and full reporting of outcomes
☐	☒	Estimates of effect sizes (e.g. Cohen's *d*, Pearson's *r*), indicating how they were calculated

Our web collection on statistics for biologists contains articles on many of the points above.

Software and code

Policy information about availability of computer code

Data collection	ANY-maze software version 5.2 (Stoelting Co)
Data analysis	GraphPad Prism 7 for Windows (version 7.04); ImageJ v1.52a; Adobe Photoshop CS6 v20.0.0

For manuscripts utilizing custom algorithms or software that are central to the research but not yet described in published literature, software must be made available to editors/reviewers. We strongly encourage code deposition in a community repository (e.g. GitHub). See the Nature Research guidelines for submitting code & software for further information.

Data

Policy information about availability of data

All manuscripts must include a data availability statement. This statement should provide the following information, where applicable:

- Accession codes, unique identifiers, or web links for publicly available datasets
- A list of figures that have associated raw data
- A description of any restrictions on data availability

All relevant data are available from the corresponding authors upon reasonable request. The source data underlying Figs 1b, d, f, 2d-g, and 3a-c and Supplementary Figs 1b, 2, 3b, d, 4b, and 5 are provided as a Source Data file.

Field-specific reporting

Please select the one below that is the best fit for your research. If you are not sure, read the appropriate sections before making your selection.

☒ Life sciences ☐ Behavioural & social sciences ☐ Ecological, evolutionary & environmental sciences

Life sciences study design

All studies must disclose on these points even when the disclosure is negative.

Sample size	Sample size is similar to samples sizes routinely used in the field for similar histological or behavioral comparisons.
Data exclusions	No data were excluded from the analyses.
Replication	Main behavioral results were successfully replicated in three independent attempts.
Randomization	All mice were allocated into their experimental groups randomly.
Blinding	Investigators were blinded to group allocation during data collection and analysis.

Reporting for specific materials, systems and methods

We require information from authors about some types of materials, experimental systems and methods used in many studies. Here, indicate whether each material, system or method listed is relevant to your study. If you are not sure if a list item applies to your research, read the appropriate section before selecting a response.

Materials & experimental systems

n/a	Involved in the study	
☐	☒	Antibodies
☒	☐	Eukaryotic cell lines
☒	☐	Palaeontology
☐	☒	Animals and other organisms
☒	☐	Human research participants
☒	☐	Clinical data

Methods

n/a	Involved in the study	
☒	☐	ChIP-seq
☒	☐	Flow cytometry
☒	☐	MRI-based neuroimaging

Antibodies

Antibodies used	Rabbit polyclonal anti-c-Fos antibody (1:1000 in PBST; Santa Cruz Biotechnology, California); Alexa Flour 594-conjugated donkey anti-rabbit secondary antibody (1:500 in PBST; Invitrogen); Alexa Flour 488-conjugated donkey anti-rabbit secondary antibody (1:500 in PBST; Invitrogen).
Validation	All antibodies were used previously in other studies and validated by the manufacturing company.

Animals and other organisms

Policy information about studies involving animals; ARRIVE guidelines recommended for reporting animal research

Laboratory animals	8-40 week old male FosCreER mice obtained from the Jackson Laboratory (Jackson lab stock#: 021882) and bred in-house at the Biological Sciences Facility were used for all experiments. Animals were group-housed in a temperature- and humidity-controlled room on a 12 h light/dark cycle with ad libitum access to food and water.
Wild animals	The study did not involve wild animals.
Field-collected samples	The study did not involve field collected animals.
Ethics oversight	All procedures were performed in accordance with the guidelines of the Canadian Council on Animal Care and the University of Toronto Animal Care Committee.

Note that full information on the approval of the study protocol must also be provided in the manuscript.
